# Potentiality of Nanoenzymes for Cancer Treatment and Other Diseases: Current Status and Future Challenges

**DOI:** 10.3390/ma14205965

**Published:** 2021-10-11

**Authors:** Rakesh K. Sindhu, Agnieszka Najda, Prabhjot Kaur, Muddaser Shah, Harmanpreet Singh, Parneet Kaur, Simona Cavalu, Monika Jaroszuk-Sierocińska, Md. Habibur Rahman

**Affiliations:** 1 Chitkara College of Pharmacy, Chitkara University, Punjab 140401, India; rakesh.sindhu@chitkara.edu.in (R.K.S.); prbhjotkaur45@gmail.com (P.K.); harmanaulakh099@gmail.com (H.S.); parneetdhaliwal11@gmail.com (P.K.); 2 Department of Vegetable Crops and Medicinal Plants, University of Life Sciences in Lublin, 50A Doświadczalna St., 20-280 Lublin, Poland; 3 Department of Botany, Abdul Wali Khan University Mardan, Mardan 23200, Pakistan; 4 Faculty of Medicine and Pharmacy, University of Oradea, 410087 Oradea, Romania; simona.cavalu@gmail.com; 5 Institute of Soil Science and Environment Shaping, University of Life Sciences in Lublin, 7 Leszczyńskiego St., 20-069 Lublin, Poland; monika.jaroszuk@up.lublin.pl; 6 Department of Pharmacy, Southeast University, Banani, Dhaka 1213, Bangladesh; 7 Department of Global Medical Science, Wonju College of Medicine, Yonsei University, Wonju 26426, Gangwon-do, Korea

**Keywords:** nanozymes, nanomaterials, artificial, cancer diagnosis, therapeutics, biomedical

## Abstract

Studies from past years have observed various enzymes that are artificial, which are issued to mimic naturally occurring enzymes based on their function and structure. The nanozymes possess nanomaterials that resemble natural enzymes and are considered an innovative class. This innovative class has achieved a brilliant response from various developments and researchers owing to this unique property. In this regard, numerous nanomaterials are inspected as natural enzyme mimics for multiple types of applications, such as imaging, water treatment, therapeutics, and sensing. Nanozymes have nanomaterial properties occurring with an inheritance that provides a single substitute and multiple platforms. Nanozymes can be controlled remotely via stimuli including heat, light, magnetic field, and ultrasound. Collectively, these all can be used to increase the therapeutic as well as diagnostic efficacies. These nanozymes have major biomedical applications including cancer therapy and diagnosis, medical diagnostics, and bio sensing. We summarized and emphasized the latest progress of nanozymes, including their biomedical mechanisms and applications involving synergistic and remote control nanozymes. Finally, we cover the challenges and limitations of further improving therapeutic applications and provide a future direction for using engineered nanozymes with enhanced biomedical and diagnostic applications.

## 1. Introduction

**Figure 1:**
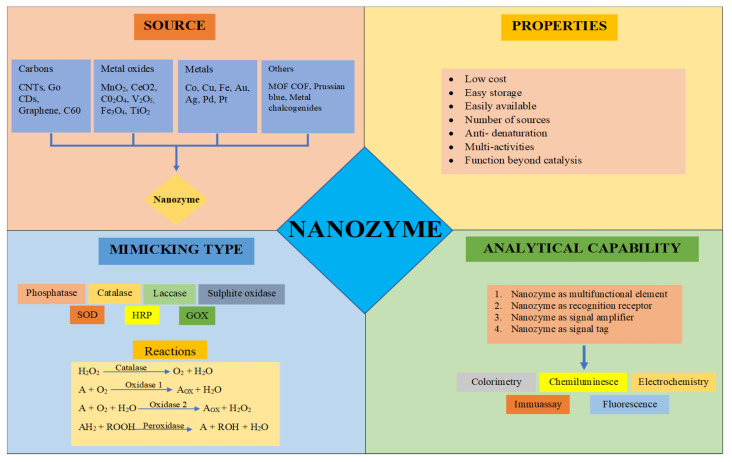
Sources, properties, mimicking types, and analytical capabilities of nanozymes.

Enzymes are considered natural biocatalysts which catalyze many biochemical reactions with good catalytic efficiency, biocompatibility, and substrate specificity. Recently, these reactions have been extensively used in various food industries and other biomedical applications. Their use in the agri-food industry promotes proper processing, storage activities and the functionalization of food products [1,2,3,4,5,6]. Enzymes play a significant role in enhancing the safety of food products [7]. Nanotechnology is believed to have a major part in advanced drug formulation, targeting a specific part of the body and controlled release of the drug. Nanotechnology is stated to communicate with the barrier of physical and organic sciences by putting forward nanospheres and structures in numerous scientific fields [8,9] other than nanomedicines and their delivery [10,11]. Nanotechnology engages the therapeutic agents at nanoscale levels for the development of medicines that are nano. Biomedicine including nanobiotechnology, biosensors, and tissue designing is done by the nanoparticles [12]. Recently, nanomedicines have become very much refreshing as nanostructures act as delivery agents by giving medication examples [13,14]. Using conveyance nano-drugs for the treatment depends upon various properties of targeted drugs such as biochemical functions [15]. Over the past few years, scientists have made an extraordinary attempt in developing artificial enzymes for various types of applications. Consider the examples that the chemical complexes based on porphyrin [16,17], hematin [18], cyclodextrin [19], hemin [20,21], and the specially designed biomolecules proteins successively imitate the function of the naturally occurring enzymes [22,23].The intrinsic limitations of the natural enzymes such as low stability, high cost, and storage difficulty have led to the introduction of artificial enzymes that imitate the activity of the naturally occurring enzymes [24]. As another sort of promising artificial enzyme, nanozymes have demonstrated a wide range of uses because of their evident favorable circumstances, including low cost, high stability, the large surface area for functionalization, high catalytic activity, and tuneable activity [25]. Various obstacles and constraints of further developing therapeutic applications are of significant interest, as well as a future direction for the usage of modified nanozymes with better biomedical and diagnostic applications. Nanozymes are defined as artificial nanomaterials possessing intrinsic enzyme-like activities. Scientists have worked toward their enhancing utility as they have many advantages over natural enzymes. Nanozymes are believed to act by mimicking the action of the natural enzymes [26,27,28]. The concept of nanozyme has reformed our essential comprehension of chemistry and biology, encouraging plenty of uses in the fields of biosensing, science, and medication [26]. Nanozyme synthesis is an innovative technology since it connects nanoparticles with biological activities and framework. Various assays have been implemented for the enzymes of proteins that also implement nanozymes, which could also have the potential for performing the catalysis of similar substrates. Due to such different functions of nanozymes, they are used for the treatment of the environment, biosensing, agents that act against microbes, cytoprotection of different cell biomolecules with management, diagnosis of diseases, etc. [29,30,31,32,33]. Various sources, properties, mimicking types, and analytical capabilities are shown in Figure 1 [1].

## 2. Types of Nanozymes

The nanozymes can be classified into different types (Table 1) based on the enzymes whose actions they mimic. It was mentioned in a 2013 review that there are generally four types of redox enzymes which include catalase, superoxide dismutase (SOD), oxidase, and peroxidase [34].

### 2.1. Peroxidasemimics

#### 2.1.1. Iron-Based

The Fe_3_O_4_ NPs, which are magnetic, have functions such as imitating the intrinsic peroxidase, which was discovered in 2007 by the Yan group. It could lead to the oxidation of the three colorless peroxidase substrates involving ortho-phenylenediamine, TMB, and diazoamino benzene to the colored materials, which are comparable with H_2_O_2_. The MNP nanozymes have the occurring mechanism, which is known as ping pong catalysis, and is suggested in the kinetics studies. The higher as well as lower affinity of nanozymes towards TMB and H_2_O_2_, respectively, compared to the HRP, has been well indicated by the measured Michaelis–Menten constants [35]. Later on, peroxidase imitates linked with Fe_3_O_4_ MNP were applied for detecting glucose and hydrogen peroxide [35,36].

#### 2.1.2. Vanadium-Based

The first demonstration regarding the V_2_O_5_ nanowire-based peroxidase mimics was done by a group named Tremel in 2011 [37]. After that, maximum attention was gained by the peculiar vanadium haloperoxidase imitating the functions of V_2_O_5_ wires which are nano along with their anti-biofouling and marine applications [38]. Various research on vanadium disulfides and peroxidase, which is similar to vanadium, has been reported since then [39,40,41,42,43].

#### 2.1.3. Based on Noble Metal

There are various nanomaterials based on noble metals such as gold [44,45,46,47,48,49,50,51,52,53,54,55], silver [56,57,58,59,60,61], platinum [62,63,64,65,66,67,68,69,70,71,72,73,74,75], Pd [76,77,78], and multi-metallic NPs which are known as peroxidase imitates and are utilized for antibodies, therapy, and biosensing.

#### 2.1.4. Carbon-Based

Carbon is another typical nanomaterial as peroxidase-like activities with pH, temp, and hydrogen peroxide concentration dependent functions have been possessed by nanotubes which have a single wall and oxides of graphene [79,80]. Propelled by these findings, there are various other carbon-based peroxidase mimics such as carbon dots [81,82,83,84,85,86,87,88], Fe/N doped carbon [89,90,91,92,93,94], carbon nitrides [95,96,97], etc., which have been explored.

#### 2.1.5. Based on Metal–Organic Framework

This framework which has diverse porous structures has been used widely for biomedical applications. It can consist of coordinating ions or clusters of metals (e.g., Cu and iron) comprising the organic ligands. 2D MOFs are believed to exhibit high functions of catalysis as compared to the analogues of 3D, hence giving effective sensibility for the detection of biomolecules [98].

### 2.2. Oxidase Mimics

#### 2.2.1. Gold-Based

Even though the nanomaterials, which are metals, are generally utilized for showing catalyzing responses, the disclosure of carbon-upheld Au or unbearable citrate-covered AuNPs (along with 3.5 nm of normal distance) and glucose oxidase-copying exercises were amazing and startling [99,100]. Further, according to the kinetics measurements, the mechanism of EleyRideal was suggested for AuNP-based imitates of oxidase [101].

#### 2.2.2. Copper-Based

Nanoparticles that contain copper were have also been used as imitates of oxidase. For example, Goximitating composites of Cu_2_O or polypyrrole were accounted for the oxidative catalysis of glucose for creating hydrogen peroxidase in fundamental terms. Although oxidation of rising glucose exercises the composites of Cu_2_O or polypyrrole, which guarantee identification of glucose, the situation for this response ought to be additionally improved in terms of physiological conditions for more extensive utilizations [102].

#### 2.2.3. Molybdenum-Based

It has been reported by Tremeland co-workers that the molybdenum trioxide nanoparticles can mimic sulfite oxidase for converting it into sulfate [103]. The high stability in water and serum led to the development of ultra-smallMoO_3_ NPs (with 2 nm average diameter).

#### 2.2.4. Based on Platinum

An important part is played by ferroxidases generally in the transfer and storage of Fe in the cellular environment. Later on, some research linked with PtNPs as the imitates of ferroxidase for oxidizing them were noted. Examples such as Zhang, Knezand collaborators used apoferritin, which is a light chain as the platform for PtNPs to get ready. Nanozymes such as these organized one scan control the homeostasis of the iron cell, which is profited by the ferroxidase [104].

### 2.3. Catalase Mimics

Catalase is believed to decompose H_2_O_2_ into H_2_O and O_2_ effectively. Many nanomaterials such as metal oxides, metals and PB exhibit the type of activities linked with catalase [105,106,107,108,109,110,111,112]. It was noted that nanoparticles had activities similar to catalase with another catalyst-impersonating function, and pH or temp could have made the catalyst impersonating action predominant. Pt and Pd were demonstrated for possessing the good imitating functions of catalase compared to gold and silver [113]. Some metal oxide nanomaterials (such as ZrO_3_ and CoO_4_) and PB were also found to show catalase-mimicking properties at higher pH [114,115].

### 2.4. Superoxide Dismustase (SOD) Mimics

The damage that occurred through oxidation to the living organization may be caused by the species of dysregulated oxygen which is reactive. Naturally, SOD is believed to eliminate the anion which is superoxide O_2_^−^, ROS, throughout the dismutation response of O_2_^−^ to hydrogen peroxide and oxygen. For surviving constraints linked with SOD, which is common, an assortment linked with nanomaterials is utilized to imitate SOD [116,117,118,119,120,121,122,123,124].

#### 2.4.1. Carbon-Based

C_60_[C(COOH)_2_]_3_, comprised of symmetry linked with C_3_, has been approved for possessing more properties such as acting against oxidation [125]. The catalytic elimination of the superoxide anion O_2_^−^ resulted in the antioxidation activity. Later on, the non-change of C_60_-C_3_ and production of O_2_ and H_2_O_2_ from O_2_^−^ was confirmed by the mechanism studies just like the SOD catalyzed reaction [126].

#### 2.4.2. Cerium-Based

Nanoceria was classified as one of the first nanomaterials possessing SOD-mimicking activity. These have been allocated to the shuttle of electrons between the mixed states of oxidation [127,128]. The superoxide mechanism of action showing the cerium oxide’s ability to scavenge has yet not been verified, but there are some studies showing more SOD-mimicking activity by the high ratio of Ce^3+^/Ce^4+^ [129,130,131].

#### 2.4.3. Melanin-Based

The nanoparticles which are melanin in nature comprise various free radicals which scavenged the activities which have been implemented by a group of Shi [132]. The mixture of the hydrochloride of dopamine along with NH_3_ in the ethanol and water led to the synthesis of melanin nanoparticles. Further, their stability was improved by functionalizing with amine-terminated PEG. Such types of PEGcMeNPs with an approximate diameter of 120 nm were shown to possess SOD-like activities for O_2_^−^ scavenging.

### 2.5. Hydrolase Mimics

The hydrolysis of the chemical bond is catalyzed by hydrolase. For example, the bonds of nucleotides are hydrolyzed by nucleosidase. The phosphatase enzyme helps in the catalysis of phosphate cleavage from the molecules [24].

#### 2.5.1. Carbon-Based

Other than the previously mentioned peroxidase and SOD-imitating activity, ultimately, nanozymes that are based on carbon are initially found to replicate the common nucleases [133]. Aqueous-solvent fullerene worked with the corrosive carboxylics, known as C_60_-1, and it was exhibited for catalyzing the phosphodiester cleavage obligation occurring in DNA and illuminated through the light. Hence, by formulating the fullerenes and the corresponding DNA, the effectiveness of a particular DNA’s cleavage site would be increased [134,135]. Notwithstanding fullerenes, the oxides of graphene were additionally utilized as hydrolase imitates [136,137,138].

#### 2.5.2. Monolayer Functionalized AuNP-Based

AuNPs worked along with monolayers which are catalytic throughout the bonds of gold and silver are amongst the very first nanomaterials imitating as hydrolases that deserve acknowledgments. According to further studies, such performance has been allocated to increased common HPNP concentration, the cooperation between more than one center of metals and the stability being high [139,140,141].

#### 2.5.3. Metal–Organic Framework-Based

Countless MOFs based on Zr are used as imitates of phosphor triesterase for the occurrence of cleavage of the bond of phosphate ester of CWA which is abbreviated as chemical warfare agent [142,143,144,145,146,147,148,149,150]. This was because of the similarity between their structures.

### 2.6. Other Enzyme Mimics

Other than hydrolysis and redox reactions, many reactions attained huge importance [151,152,153,154,155,156,157]. For example, other than peroxidase and the imitates of hydrolase discussed prior, an action like hydrogenase would likewise be figured out, insofar as giving MOFs photon ingestion specialists (porphyrin) and proton ingestion lessening operators (PtNPs) [158,159,160]. Moreover, the synthesis of MOFs with carbonic anhydrase limits the dangerous atmospheric deviation issue [161].

Furthermore, Chmielewski et al. revealed that the assembly of electrostatics, the peptide parts of trimethylammonium working AuNPs, could advance the ligating of peptides that are two in number, which resulted in inorganic implemented nanoparticles favorable in the biopolymers polymerization [162]. Morse et al. also illustrated the monolayer AuNPs functionalization which could mimic silicatein.

#### 2.6.1. Single-Substrate Mechanism of Nanozymes

Such types of nanozymes only show a reaction with one substrate. Certain nanozymes were revealed by imitating the action of enzymes while giving a platform which is water-soluble. Basic functionalized groups are moored with cooperation onto different stages of nanoscale for synergist responses. Afterward, multivalent components such as metal ions came into consideration and have developed, expanding utilization in the biomedical field. Representative nanozymes along with the one-substrate mechanism would be sorted into a few types, dependent on responses and the sorts of enzymes occurring naturally.

This mechanism usually displays the kinetic profile which shows catalysis by Michaelis–Menten, in which catalysis has two stages, that is, the authoritative and response stages. While plotting the velocity of the reaction as a function of the concentration of substrate, kcatand KM are commonly determined to characterize nanozyme movement.

The nanozymes showing a single substrate mechanism generally include:

- Hydrolase;
- Peroxidase;
- Superoxide dismutase;
- Oxidase;
- Catalase [26].

#### 2.6.2. Nanozymes with the Multi-Substrate Mechanism

The advancement of nanotechnology and the comprehension of artificial enzymes have amassed, and their mechanism of multiple enzyme-like activities has been recognized. Representative nanozymes that follow one or more substrates or work differently under different situations, such as pH esteems, hydrogen peroxide or glutathione concentrations, and oxygenation levels, are listed in this section. These components may altogether impact the practices of nanozymes, which are particularly valid for the organic microenvironment at disease sites like cancer.

The multi-substrate mechanism can be depicted in:

- Metal-based nanozymes;
- Cu_2_O nanozymes;
- Nanoceria;
- Melanin nanoparticles;
- Prussian blue nanoparticles [26].

## 3. Synthesis of Nanozymes

### 3.1. Nanozyme Production

The nanomaterials which are catalytic possess different properties in comparison with natural enzymes [163]. The activities of the nanozymes depend on the size of the particle, structure, and its shape which is affected by the coatings, charges, and external fields [164,165].

### 3.2. Hydrothermal and Solvothermal Methods

The techniques which are very promising for synthesizing the nanomaterials are the hydrothermal and solvothermal methods. Nanocrystals of low cost with well-controlled dimensions can be obtained by utilizing the proposed methods [164,166].

A series of nano crystals which are spinel-type were synthesized by using the method of solvothermal, where the solvent used was ethylene glycol. The obtained nanozymes were utilized as enzyme mimics for the detection of hydrogen peroxide. For example, two types of carbon-based nano catalysts with a size of 100–150 nm were synthesized by utilizing a combination of two methods, a thermal method and a solid-state reaction, from the zeolitic imidazolate framework-8 (ZIF-8) [167,168]. The carbon cubic nanomaterial with the hollow structure was procured by chemically etching ZIF-8 along with tannic acid, stuck to it by a calcination process. The carbon cubic nanomaterial with the porous surface was acquired by direct pyrolysis [169].

Electrochemical observation of glucose and fructose formed on gold nanoparticles (AuNPs) placed onto graphene paper has lately been presented. These nanostructures were formed by two techniques: thermal and laser de-wetting processes [170]. Gold nanostructures acquired by both methods exhibited major differences in their particle morphology. Both types of AuNPs were investigated by their capacity to oxidize glucose and fructose [171].

### 3.3. Chemical Reduction

Chemical reduction is a method which is used very frequently because of its rapidity and simplicity. This tool enables the formation of NPs in which the morphology and the size of particle distribution are managed by changing the molar concentration of the reactants, the reductant type, and the reaction temperature [172]. The important factor in achieving very high chemical reduction is choosing the suitable reductants. The reduction of metal salts needs reactivity of the agent which causes reduction to the redox potential of the metal. The procured particles are small if the reaction rate during the synthesis procedure is too fast [173]. Nevertheless, if the reaction rate is too slow, particle aggregation may happen [174]. The synthesis of hollow copper sulfidenanocubes (h-CuS NCs) was done via the chemical reduction method [175]. This method has been utilized for the synthesis of peroxidase (PO)-like nanozyme-based AuNPs along with Pseudomonas aeruginosa-specific aptamer [176].

### 3.4. Sol–Gel Method

In the sol–gel method, a gel-like network containing liquid and solid phase is formed. The crystallinity, morphology, and magnetic properties of the nanozymes can be managed by choosing a suitable complexing agent, concentration and type of chemical additive, and temperature conditions [177]. The synthesis of PtNPs polyaniline (PAni) hydrogel heterostructures was produced with the sol–gel method [178]. Phytic acid was utilized as a complexing agent. The PtNPs loaded into the hydrogel matrix act as active catalysts for the oxidation of hydrogen peroxide. The acquired PtNP/PAni hydrogel had a 3D hierarchical structure consisting of connected PAni nanofibers with diameters of approximately 100 nm). The porous structure of the PAni hydrogel allows immobilization of concentrated enzyme solutions. Since water-soluble molecules can penetrate through the hydrogel, the PtNPs preserve their ability to catalyze glucose oxidation [179].

### 3.5. Co-Precipitation

Co-precipitation is a quick technique for the amalgamation of various sorts of nanocatalysts. Co-precipitation is a superb method to use when higher virtue and better stoichiometric control arerequired. Dashtestani et al. utilized a mix of two strategies for nanocompositeunion: reduction of HAuCl4 chemically and co-precipitation of the acquired gold nanoparticles with the copper (II) complex of cysteine (GNPs/Cu-Cys). The mix of GNPs and the Cu-Cyscomplex expanded the electrochemical sign toward O_2_ [180,181].

### 3.6. Electrochemical Deposition

Electrochemical deposition is a minimal-effort strategy for acquiring metalnanocatalysts. In any case, it is normally utilized less regularly than synthetic decrease strategies. The interaction is straightforward and incorporates a drenching of a conductive surface into an answer containing particles of the material to be saved and the use of a voltage across the strongelectrolyte interface. Throughout this strategy, a reaction of charge transfer causes the deposition of film [182,183].

### 3.7. Polymerization and Polycondensation

Nanozymes can be acquired either by utilizing insoluble polymers or by cross-connecting of a solvent polymer. Santhosh et al. blended composite center shell nanofibers comprising gold nanopartilces on poly (methylmethacrylate) by the mix of an electrospinning procedure, and furthermore, the in situ polymerization of aniline [184,185].

## 4. Nanozymes from Challenges to Opportunities

**Figure 2:**
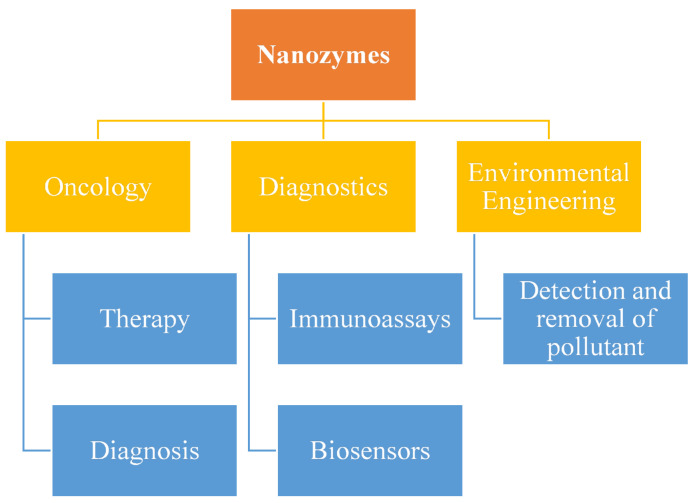
Opportunities in the field of nanozymes.

There has been a huge development observed in the field of nanozymes over the last few years, as shown in Figure 2. There has even been a considerable increase in the number of nanozymes along with the reaction. Enzymes are found to depict some of the basic characteristics such as high substrate specificity and excellent activity. Besides this, there are a variety of nanozymes which did not achieve the level of composure. Such limitations are served as a better opportunity for advanced development and research [186].

Types of Nanozymes

Many studies are describing different types of nanozymes (e.g., Fe_3_O_4_ nanoparticles were observed to possess peroxidase-like activity), indicating their ability to catalyze the substrate oxidation by using hydrogen peroxide [187,188]. Due to the biocompatible and magnetic qualities of Fe_3_O_4_, it is interesting and can be used in theragnostic in vivo procedures [189]. Later on, it was observed that many nanoparticles possess the same peroxidase-like activity [190]. Nanoceria consists of different types of enzyme mimics such as superoxidase dismutase (SOD), oxidase, and catalase [191]. Gold NPs can mimic glucose oxidase.

### 4.1. Biosensors

Electrochemical, calorimetric, and fluorescence detecting are the traditional procedures for the evaluation of estimated constituents through a corresponding difference in organic systemization and are further broadly utilized for the recognition of biomolecules [192]. Among these, the ELISA, conventional calorimetric discovery, is utilized for distinguishing exceptionally small amounts of the wanted substance [193]. The biosensors which are based on nanozymes are effectively created to recognize ions.

### 4.2. Detection of H_2_O_2_

It is linked generally along with transduction of single and cell growth. Excessive production of H_2_O_2_ may lead to an increased risk of many inflammatory infections such as lung diseases, atherosclerosis, hepatitis, etc. [194,195]. The detecting of H_2_O_2_ is important because of its significance in the field of biology and medicine. The iron oxide MNPs working as imitates of peroxidase were used to initially detect hydrogen peroxide with the chromogenic 2,2′-azino-bis (3-ethylbenzothiazoline-6-sulfonic acid) (ABTS) [196]. Various peroxidase nanozymes have been designed for calorimetric detection of H_2_O_2_ [197,198].

### 4.3. Detection of Glucose

These have attained a lot of consideration in the last several years due to their broad employments in clinical examination, biomedical sciences, food creation, and biology [199,200]. Glucose and comparative parts that can form hydrogen peroxide by the synergist response could be detected as per particular peroxidase and oxidase mimics [201]. A method for direct physical adsorption development of electrochemical biosensing of glucose was accomplished. This planned biosensing showed selectivity which was high and had sample feasibility [200].

### 4.4. Metal Ion Sensing

Many studies focus generally on the uses for sensing [201,202]. During the underlying work around there, a particular and sensitive sensor was created to identify Cu^2+^ utilizing Cu^2+^. The magnetic and nanotube silica NPs of carbon were used to create the extremely delicate sensors to discover the exceptionally low Cu^2+^ quantity [203]. In another examination, the platinum NPs (2 nm) with cow-like serum egg whites were created to examine imitating peroxidase action. This was useful for building up specific and touchy sensation for identification of Hg which has a straight identification of 0–120 nM of range [204].

### 4.5. Nucleic Acid Sensing

A few methodologies for the recognition of nucleic acids are created by the usage of nanozymes [205,206]. A test was created to identify bacterial DNA utilizing Fe_3_O_4_ MNPs to examine the checking of microorganisms in faucet water [207]. The proclivity towards different nanozymes is distinctive for abandoned DNA which is single as well as twofold abandoned DNA. Calorimetric technique is produced for the discovery of DNA through adjustment of peroxidase mimicking movement of “Au” nanoparticles on graphene sheets [208].

### 4.6. Aptasensors

These are used for building aptasensors for the little molecules which are bioactive and proteins [209,210]. Utilizing the AuNPs having peroxidase-like activity alongside high particularity and acetamiprid’s affinity explicit S-18 aptamers, a calorimetric assay was intended for pesticide quick checking [211]. The acetamiprid presence inside the sample could communicate along the aptamer which prevents binding and also helps in recovery.

### 4.7. Pollutant Detection

Melamine, a nitrogenous natural compound that becomes toxic when taken and has been illegally used in dairy products, was identified using a fast and efficient calorimetric approach [212]. The compound is found to work by repressing the reactant ABTS oxidation by NPs with hydrogen peroxide, yet it emphatically responds with it and creates compounds. The nanozyme-based strategies have been so far successful, straight forward, and cost-accommodating for mineralizing and debasing the natural colors of mechanical strategies. Most prominently, MNPs like peroxidase were considered to debase the natural pollutants. The degrading procedure gives a recognizable advantage on degradation methodology for extraordinary strength and diminished expense. The MNPs/H_2_O_2_ can effectively achieve the evacuation of 85% of phenolic mixes from the fluid arrangement in 3 h [213]. Degradation based on MNPs showed higher viability. It was discovered that 96% of this color could be degraded inside 15 min utilizing improved conditions [214].

### 4.8. Nanozyme-Based Immunoassays

Many considerable efforts have been made for designing immunoassaying with the help of nanozymes [215,216]. Various configurations were used for immunoassays utilizing the nanozymes as the signaling parts. For example, the sandwich immunoassay and antigen down (AD) immunoassay have been revealed [217]. Later on, various researchers executed the standard sandwich immunoassay for identification by using nanozyme mimics of peroxidase and oxidase [215]. For example, the preparation of imitates of peroxidase with enhanced activity was conducted [218].

## 5. Nano-Enzymes Role in Diseases

### 5.1. Cancer Diagnostics and Therapy

To detect cancer cells, nanozymes are being implicated [219]. Various substrates that are organic undergo catalytic oxidation which has been shown by cerium oxides NPs [220]. These were introduced for the immuno detection of cancer cells regarding the unique capability of folate conjugation. According to various studies, the folate receptor which is over-expressed onto the cancer cells is being detected selectively by folate. With the dissimilarity of ELISA, immunoassays based on the oxide of cerium manifested various benefits. However, these techniques which conventionally need the support of various antibodies may have some limitations when antibodies are denatured on the cancer cell surface. Secondarily, durability-like shortcomings are exhibited. Hence, when it is denatured, it may result in losing its original catalytic activity. Many researchers have said that NPs of cerium oxide are not considered as imitates of oxidase. Regardless of this, it is assisted as the catalyst of oxidation. Further, some people learned about this by nanoprobes [221]. Other than this, the nanocomposite was introduced by nanoparticle development which is gold [199]. Peroxidase can be mimicked by the nanocomposites which are formed.

**Figure 3:**
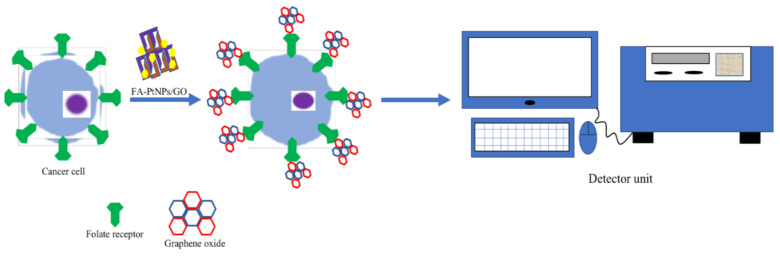
PtNPs/GO nanozymes for detecting cancer cells with calorimetric strategy.

The detection of cancer cells is implicated by nano-enzymes [219]. Figure 3 shows the detection of cancer cells with calorimetric strategy by using PtNPs/GO nanozymes. Regulation of gene process is majorly served by RNA interference [222,223]. A group introduced nanozymes which are similar to machinery, based on the structural characteristics and functional characteristics of this system which areused for the target RNA to be cleaved [224]. In this particular technique, gold nanoparticles act as the keystone of the nanozymes that offers a modification of DNA which are single-stranded oligonucleotides and endonucleases that are non-specific. The resulting nanocomposites indicated RNA-DNA nanoparticles. It was found that nanoparticles may inhibit virus replication and gene expression silencing. Diseases with expressions of proteins such as infections caused by viruses or cancers are associated with nanozymes. Photodynamic therapy employs nanozymes under hypoxia [225]. Singlet oxygen is formed from the tumor tissues by organic metal frameworks based on photodynamic therapy [226,227]. Tumor tissues are also having hypoxic conditions that are restricted by the therapeutic properties of photodynamic therapy [228]. The new technique was introduced for the photodynamic therapy through some modifications, and for this, oxygen was generated by the catalase mimics, which is PtNPs. On to the surface of organic–metal frameworks where Pt NPs are assembled, the production of oxygen which is single can be raised by the nanocomposites through the decomposition in the hypoxia’s tissue of hydrogen peroxide. Under the hypoxic environment, the cancer treatment is effectively served by nanozyme integrated organic–metal frameworks [229]. Malignant tumors are treated with certain approaches, and one of the promising approaches is apoptosis induced with nanozymes [230]. The treatment systems which are presently used require the support of ROS and oxygen under conditions of tumor. A successful nano-flower is biomimetic and was designed by assembly of various nanozymes [231]. The PtCo-NPs in the acidic tumor produce toxic ROS by oxidase mimics, whereas the MnO2 nanomaterials possess good catalase-like functions [232].

### 5.2. Neuroprotection

Various researchers have been developing the application for SOD imitates for guiding free radical destruction [233,234]. Afterwards, the C_60_[C(COOH)_2_]_3_ was introduced as an imitating agent of SOD, which shows therapeutic actions in knockout mice [235]. In SOD2 mice not able to express the manganese SOD in mitochondria, the period of life was increased. Nanoceria was introduced to imitate the SOD and also show various functions of neuroprotection [236]. Confirmation was done about nanoceria that it can do the aversion of the cells of the neurons present in the retina by the destruction of ROS [237]. In Alzheimer’s disease, RNS peptides and amyloid-beta are involved. The treatment for this disease is missing for the antioxidants. Microscopic studies suggested that the neurons internalize the nanoceria that is present. It can perform the RNS scavenging and hence results in the protection of neurons that are degenerated. Studies have suggested that nanoceria is used for the protection of the neurons from hyperphosphorylation or their death [238].

### 5.3. Antioxidation

The cell metabolism has certain by-products which are O_2_, OH^−^, and H_2_O_2_ [239,240]. The ROS contributes to various signaling mechanisms and when the level of ROS is considered low, they act as second messengers which are significant [241]. On the other hand, if the ROS levels are exceeded, then they result in the damage of proteins, DNA, lipids, and other various molecules. Moreover, for activating the apoptosis of cells, the caspase can be induced by them [242]. Various pathological disorders are linked with ROS, for example: kidney diseases, diabetes, arthritis, cancer, and atherosclerosis [243]. It is known that ROS affects human health and life critically by participating in aging, human diseases, and death. Hence, for maintaining the intracellular redox homeostasis, the levels of ROS need to be regulated majorly. Within the cell system, various types of enzymes are antioxidants, for example: catalase, glutathione peroxidase, SOD, etc. An important function is being performed by the antioxidant enzymes which are maintaining the cellular redox balance. However, ROS overexpression can decrease the activities of enzymes which are under pathological situations [244].

### 5.4. Anti-Inflammatory

Inflammation is the state which involves two types of inflammation: acute and chronic inflammation [245]. The precursor to various diseases is certainly the response of inflammation. Hence, the emergence of various associated disorders can be treated successfully by treating inflammation, for example, diseases associated with heart and cancer. The quality of increase in the ROS is notable in the tissue of inflammation. The inflammation is alleviated by scavenging ROS along with their inhibition of diseases. From the time of the formation, the Prussian blue has been working as blue dye [246]. The Prussian blue has been employed as an antidote for thallotoxicosis in clinical trials because of its brilliant biosecurity along with the biocompatibility as recommended by the FDA [247]. For the treatment of cancer, the PB-NPs pay a role in photo thermal therapy, ultrasound imaging, and magnetic resonance imaging [248,249]. The nanoparticles PB have been discovered which possesses multi-enzyme-like functions, which can successively reduce the levels of ROS which are inside the cell and also attain the cytoprotecting efficacy [250]. It was noted that the feature of scavenging ROS was due to the attraction towards the hydroxide along with the mimicking of the enzymes. The altered immune response against the infection causes organ dysfunction, which is a great threat to life known as sepsis [251]. The systemic inflammation occurred by the microbe local infection which is further accompanied by fever, and because of host defense mechanisms, white blood cells increase in number [252]. If, within a short period, the treatment is not done, then the immune response becomes disordered which would cause multiorgan dysfunction, pro-inflammatory cytokines, and even death. Hence, if we inhabit the abnormal inflammatory response, then it would kill the bacteria [253].

### 5.5. Anti-Aging

Data have suggested that aging is somewhat linked with major mechanisms of redox, such as ROS detoxifying and the response of cells to the macromolecules which are damaged oxidatively. Amice brain slice was set up for imaging, and the results showed that its treatment with nanozymes slowed down the age associated with the damage within the tested region. Using EPR techniques, it was known that the nanozymes were in action inside the powerhouse of the cell, as the primary cellular source for ROS is reported to be mitochondria. Nanozymes have an impact on age-linked loss of memory which was determined, exhibiting nanozymes’ potential for securing the age-linked cognitive damage in the mice [254].

## 6. Biomedical Application of Nanozymes

Nanozymes are found to show a greater significance in the field of biomedicine as well as industry. Numerous nanozymes have just demonstrated serious adversaries to the enzymes which are naturally occurring and which they imitate. The developing disclosure and more profound comprehension of nanozyme systems have empowered various applications linked with the biomedical industry.

### 6.1. Nanozymes Acting by Themselves

They were found in calorimetric and biosensing assays for immediate substitutes of natural enzymes. They comprised extra functions regularly which were not offered by the natural enzymes. As it is among the most utilized strategies for the detection of biomarkers, ELISA ordinarily utilizes horseradish peroxidase (HRP) for oxidizing 3,3′,5,5′-tetramethylbenzidinel (TMB) for color improvement and ensuring measurement. In any case, HRP is restricted for a scope of pH, concentration, and temp because of its instability in worse conditions and significant expense. HRP in the biosensing processes can be challenged by various categories of the nanozymes which include nanoceria, (MNPs), (GO), and other peroxidase-imitating nanozymes. Furthermore, they have invaluable natural functions that would additionally encourage research [26]. Nanozymes permit biosensing applications custom-fitted for their particular properties, giving essential refinement using attraction [255]. MoO_3_ nanozymes were discovered for the detoxification of cells done valuably by imitates of sulfite oxidase. The absence of this imitate was related to the neurological harm and youth demise [256].

### 6.2. Synergistic Nanozymes

Although nanozymes were originally found for the free action of enzyme, attempts in recent times have already been conducted more profoundly. Revising basic attributes linked with an environment, for example, H_2_O_2_ concentration, pH, and oxygenation levels, may shed light on nanozyme exhibitions. Therefore, the subsequent therapeutic and diagnostic role of nanozymes can be better adjusted.

In light of the inherent peroxidase-imitating functions of GO, Qu, and other members, a method was set up for the estimation of the concentration of glucose [80]. Zhong and others furthermore built up the catalytic cascade utilizing AuNP nanosheets; hence, the previous includes Goximitating function and can go about as a natural peroxidase [257]. MOF fills in for layout to develop and accept hydrogen peroxide.

Xia and co-workers built up the system of nanozyme which is 3 in 1, which includes detection and catalysis utilizing CD/AUNP [258]. Further, in 2017 Shi, Chan, and co-workers led to the development of therapy by using graphene oxidase for the in vitro treatment of neoplasm [259].

### 6.3. Remote Control Nanozymes

These are the types of nanozymes which are controlled and are responsible for synergistic effect.

Thus, these functions were present with accentuation in techniques for improvement concerned with the future with superb transience and exactness. Light has been described as the utilized strategy for controlling the synergist response. It may very well be delivered with high precision and controllability. Different effects of the nanozymes can be triggered by tuning the wavelength. Prinsand co-workers led to the development of light-regulating AUNP nanozymes for the hydrolysis of RNA [260].

Inflammation is commonly linked with reactive oxygen species (ROS). Propelled by common photosynthesis and the way by which hydrogen gas may lead to the reduction of ●OH to H_2_O, a multi component framework has been gathered by Chia, Sung, and co-workers to create hydrogen gas in nearness to the site of inflammation in mice [261]. Heat is considered to be another stimulant for triggering them. Nieand others built up the method of amplifying signal-free enzymes. They used Au capsules as imitates for calorimetric assay of the disease [262]. Further, Qu and co-workers designed a heat-recovering rationale entryway by utilizing nanoceria as a signal transducer [263]. Other than heat and light, nanozymes utilized ultrasound as the boost. Yeh and others detailed a hydrogen peroxide-encapsulated Fe_3_O_4_-PLGA polymer nanozyme framework for malignancy treatment [264].

## 7. Future Perspectives for Nanozymes

Intending to peruse nanozymes, one has to have a vital source of innovation through productively conquering disadvantages of enzymes which are natural, and accompanying proposals are offered. There is a requirement of the advancement of fresh nanozymes comprised of high movement and customary examination functions; further has exploration followed a technique of screening of sound action dependent on the nuclear arrangements which were conceived for catalyzing the response of enzyme. The process to prepare normal composites for identifying the present significant limitations by adjusting synergistic effects for facilitating electron transfer between composite materials during redox reaction has also been started. Bioinspired synthesis of nanozymes additionally gives an alternative to prepare non-toxic nanozymes by successfully going around the utilization of poisonous synthetic compounds in traditional substance combination, accordingly quickening their use in therapeutic application. At last, the turn of events of novel surface designing innovation could specifically target the substrates by nanozymes and would be of great significance [23]. More of these developments would open up new avenues for single-stage sensors and theragnostic, which could be helpful in various biosensing and biomedical applications. The vast majority of the nanozymes are accounted for to show their synergist movement by redox action by surface iotas. Be that as it may, the reactant movement might be additionally improved by controlling the center of the nanozymes by doping with some uncommon earth components. Such procedures would add more redox “problem areas” for synergist action and along these lines upgrade the action of nanozymes. In contrast to characteristic catalysts, the size and synthesis of most nanozymes are not uniform, except for fullerene-based nanozymes. Further, group to-cluster variety fit as a fiddle of nanoparticles/nanozymes, and consequently adjustments in physicochemical properties, requires expanded spotlight on improving the union convention to create the monodispersed nanozymes with molecularly exact designs [264].

## 8. Conclusions

The enzyme-mimicking properties of nanoparticles have proved to be significant in medicine, industry, and healthcare. Certain types of nanozymes such as peroxidase mimics, superoxide mimics, catalase mimics, etc., have contributed to various applications and emerging opportunities. Different mechanisms of nanozymes such as single substrate and multi substrate have been studied. Nanozymes were found to have various options in the field of biosensors, apt sensors, glucose and hydrogen peroxide detection, nanozyme based immunoassay, etc. Other than this, nanozymes have also played a great role in the healthcare system in cancer diagnosis and treatment, anti-aging, neuroprotection, etc. Further, biomedical applications such as self-acting nanozymes, synergistic nanozymes, etc., have also been considerably studied, which lead to various therapeutic effects.

## Figures and Tables

**Table 1:** Classification of nanozymes.

Sl. No	Nanozymes	Subtypes	Features	Reference
1.	Peroxidase mimics	Iron-based	The Fe_3_O_4_ NPs, which are magnetic, have functions such as imitating the intrinsic peroxidase. The MNP nanozymes have the occurring mechanism which is known as ping pong catalysis and is suggested in the kinetics studies.	[35]
		Vanadium-based	V_2_O_5_ nanowire-based peroxidase mimics were the first demonstration done. Other research included the peculiar vanadium haloperoxidase imitating the functions of V_2_O_5_ wires which are nano, along with their anti-biofouling and marine applications.	[37,38]
		Based on noble metal	Multi metallic NPs of the noble metals (Pd,Ag,Pt) which are known as peroxidase imitates and are utilized for antibodies, therapy, and biosensing.	[44,45,46,47,48]
		Carbon-based	They possess pH, temp and hydrogen peroxide concentration dependent functions. These have been possessed by nanotubes which have a single wall and oxides of graphene.	[79,80]
		Based on metal–organic framework	2D MOFs are believed to exhibit high functions of catalysis as compared to the analogs of 3D, hence giving effective sensibility for the detection of biomolecules.	[98]
2.	Oxidase mimics	Gold-based	According to the kinetics measurements, mechanism of Eley–Rideal was suggested for AuNP-based imitates of oxidase.	[101]
		Copper-based	Nanoparticles that contained copper were also used as imitates of oxidase. For example, Goximitating composites of Cu_2_O or polypyrrole were accounted for the oxidative catalysis of glucose for creating hydrogen peroxidase in fundamental terms.	[102]
		Molybdenum-based	It has been reported that the molybdenum trioxidenanoparticles can mimic sulfite oxidase for converting it to sulfate beneath the terms of physiology.	[103]
		Based on platinum	Some research linked with PtNPs as the imitates of ferroxidase for oxidizing them were noted. Examples such as, Zhang, Knez, and collaborators used apoferritin which is a light chain as the platform for PtNPs to get ready.	[104]
3.	Catalase mimics	--------------	There are many nanomaterials such as metal oxides, metals, and PB which exhibit the type of activities linked with catalase. Pt and Pd were demonstrated for possessing the good imitating functions of catalase compared to those of gold and silver.	[105,106,107,108,109,110,111,112,113]
4.	Superoxide dismutase (SOD) mimics	Carbon-based	C_60_[C(COOH)_2_]_3_ comprised of symmetry linked with C_3_ has been approved for possessing more properties such as acting against oxidation.	[125]
		Cerium-based	Nanoceria was classified as one of the first nanomaterials possessing SOD mimicking activity. These have been allocated to the shuttle of electrons between the mixed states of oxidation.	[127,128]
		Melanin-based	The mixture of the hydrochloride of dopamine along with NH_3_ in the ethanol and water led to the synthesis of melanin nanoparticles.	[132]
5.	Hydrolase mimics	Carbon-based	Aqueous-solvent fullerene worked with the corrosive carboxylic, known as C 60-1, and it was exhibited for catalyzing the phosphodiester cleavage obligation occurring in DNA and illuminated through the light.	[134,135]
		Monolayer functionalized AuNP based	AuNPs worked along with monolayers, which are catalytic throughout the bonds of gold and silver, are amongst the very first nanomaterials imitating as hydrolases that deserve acknowledgments.	[139,140,141]
		MOF-based	MOFs based on Zr are used as imitates of phosphor triesterase for the occurrence of cleavage of the bond of phosphate ester of chemical warfare agent.	[142,143,144,145,146,147,148,149,150]

## Data Availability

Not applicable.
